# Identification of miRNAs related to osteoporosis by high-throughput sequencing

**DOI:** 10.3389/fphar.2024.1451695

**Published:** 2024-08-08

**Authors:** Jiachun Huang, Haolin Yang, Shuang Chai, Yanping Lin, Zhihai Zhang, Hongxing Huang, Lei Wan

**Affiliations:** ^1^ The Third Affiliated Hospital of Guangzhou University of Chinese Medicine, Guangzhou, Guangdong, China; ^2^ Guangzhou University of Chinese Medicine, Guangzhou, Guangdong, China

**Keywords:** osteoporosis, microRNA, bone, high-throughout sequencing, qRT-PCR

## Abstract

**Background:**

Osteoporosis is a major health issue. MicroRNAs (miRNAs) play multiple roles in regulating cell growth and development. High-throughput sequencing technology is widely used nowadays.

**Objective:**

To screen for and validate miRNAs associated with osteoporosis.

**Method:**

Bone specimens from patients with (n = 3) and without (n = 3) osteoporosis were collected. High-throughput sequencing was used to screen for miRNAs that were then analyzed using volcano maps, Wayne maps, gene ontology (GO) analysis, and Kyoto Encyclopedia of Genes and Genomes (KEGG) analysis. Confirmation of the miRNAs was done using qRT-PCR.

**Results:**

The analysis of sequencing showed that there were 12 miRNAs that were down-regulated and five miRNAs that were upregulated in osteoporosis. GO and KEGG identified these miRNAs as being associated with bone metabolism. The qRT-PCR results showed that miR-140-5p, miR-127-3p, miR-199b-5p, miR-181a-5p, miR-181d-5p, and miR-542-3p exhibited a decrease of 2.27-, 3.00-, 3.48-, 2.67-, 2.41-, and 1.98-fold (all *P* < 0.05) in osteoporosis compared to controls. Conversely, miR-486-3p and miR-486-5p demonstrated an increase of 2.17- and 3.89-fold (*P* < 0.05) (all *P* < 0.05).

**Conclusion:**

This study utilized high-throughput sequencing to detect miRNAs that were expressed differently in individuals with osteoporosis. In osteoporosis, six miRNAs (miR-140-5p, miR-127-3p, miR-199b-5p, miR-181a-5p, miR-181d-5p, and miR-542) were found to have decreased expression, whereas two miRNAs (miR-486-3p and miR-486-5p) were found to have increased expression. The initial manifestation of various miRNAs might serve as predictive indicators and potentially anticipate the progression of osteoporosis.

## 1 Introduction

Osteoporosis, a condition affecting the skeletal system, is characterized by decreased bone density and bone structural degradation, which weakens bones and increases fracture susceptibility (LeBoff et al., 2022; Fuggle et al., 2024). The occurrence of osteoporosis is widespread, affecting approximately 20%–40% of women after menopause and 6%–8% of men aged 50 years or older (LeBoff et al., 2022; Fuggle et al., 2024). Due to the elevated fracture risk (Kanis et al., 2019; LeBoff et al., 2022; Fuggle et al., 2024), the personal and financial implications of osteoporosis hold significant importance. Estimates predict that osteoporosis-related fractures will double by 2035, reaching 5.99 million fractures and incurring a cost of 25.43 billion dollars in China by 2050 (Si et al., 2015). The healthcare burden caused by hip fractures is particularly high due to their association with significant disability and increased mortality rates, especially within the first year following the fracture (Guzon-Illescas et al., 2019). Hence, it is crucial to promptly enforce economical intervention measures in order to mitigate the societal consequences of osteoporotic fractures.

Genomics and transcriptomics heavily rely on high-throughput sequencing technology, which enables the sequencing of complete genomes and transcriptomes, making it an indispensable tool. MicroRNAs (miRNAs) are small molecules found in higher eukaryotes (O’Brien et al., 2018; Gebert and MacRae, 2019). miRNAs inhibit protein synthesis by binding to their target mRNA and directing the RNA-induced silencing complex (RISC) (O’Brien et al., 2018; Gebert and MacRae, 2019). The conservation of miRNAs during species evolution is significant (O’Brien et al., 2018; Gebert and MacRae, 2019). Specific tissues and developmental stages exclusively express miRNAs discovered in plants, animals, and fungi (Alberti and Cochella, 2017). Multiple investigations have analyzed the miRNAs associated with osteoporosis, yet they have produced contradictory findings (Gu et al., 2019), primarily due to variations in technologies, populations, or the limited focus on specific miRNAs.

The study utilized high-throughput genetic sequencing to analyze the transcriptome in the bones of three individuals diagnosed with age-related osteoporosis and three individuals without osteoporosis. The aim was to identify the particular miRNAs associated with osteoporosis.

## 2 Materials and methods

### 2.1 Patients

Specimens from the femoral intertrochanteric area or vertebrae were obtained from six orthopedic surgery patients at Guangzhou University of Chinese Medicine Affiliated Orthopedic Hospital (Table 1). All subjects were admitted to the hospital for treatment of fragility fractures and agreed to participate in this trial during their course of treatment. There were five individuals who had a background of persistent pain in the lower back and legs. Three individuals were found to have osteoporosis when their bone mineral density (BMC) or bone mineral density (BMD) levels were more than 2.5 standard deviations below the norm for healthy adults. The newly collected specimens were quickly treated with liquid nitrogen within 15 min of being harvested and then preserved in an RNA Fixer Reagent (Bioteke, Beijing, China) at a temperature of −80°C until the RNA extraction process. The research received approval from the Ethics Committee of The Third Affiliated Hospital, which is associated with Guangzhou University of Chinese Medical (No. 20200821002). All patients provided written consent.

**TABLE 1:** Characteristics of the study subjects.

Sample #	Sex	Age (years)	Height (cm)	Weight (kg)	Lumbar T-score
OP-1	Male	43	178	80	−3.4
OP-2	Female	52	156	58	−3.7
OP-3	Female	68	152	46	−2.8
NOP-1	Male	33	176	78	−1.2
NOP-2	Male	30	167	63	1.4
NOP-3	Male	60	170	66	1.4

### 2.2 RNA isolation

Novogene Co., Ltd. (Beijing, China) conducted the measurement and assessment of RNA quantity and quality. The examination of RNA degradation and contamination took place on agarose gels with a concentration of 1%.The purity of RNA was assessed by employing a Nano Photometer^®^ spectrophotometer (Implen HmbH, Munchen, Germany). The concentration of RNA was determined by utilizing a Qubit^®^ RNA Assay Kit with a Qubit^®^ 2.0 Fluorometer (Life Technologies Co., Grand Island, NY, United States). The quality of the RNA was assessed by employing the RNA Nano 6000 Assay Kit.

### 2.3 Library preparation for small RNA sequencing (Novogene Co., Ltd.)

The small RNA library was constructed using 3 μg of total RNA per sample. The NEBNext Multiplex Small RNA Library Prep Set for Illumina (New England Biolabs, Beverly, MA, United States) was utilized to create the sequencing libraries, in accordance with the guidelines provided by the manufacturer. Index codes were incorporated to assign sequences to individual samples. The 3′ SR adapter was directly and exclusively joined to the 3′ terminus of the miRNA, siRNA, and piRNA. Following the 3′ligation process, the SR RT primers were paired with the surplus of 3′SR adaptor (that remained unbound subsequent to the 3′ligation process), thereby converting the single-stranded DNA adaptor into a double-stranded DNA molecule. Preventing adaptor-dimer formation is crucial at this stage. Furthermore, dsDNAs do not serve as substrates for ligation facilitated by T4 RNA Ligase 1, consequently preventing their ligation to the 5′ SR adaptor during the subsequent ligation process. The adapter at the 5′-end was connected to the 5′ ends of the miRNAs, siRNA, and piRNA. Using the M-MuLV Reverse Transcriptase (RNase H), the initial cDNA strand was synthesized. The process of PCR amplification involved the utilization of LongAmp Taq 2× Master Mix, SR Primer for Illumina, and index (X) primer. After running at 100 V for 80 min, the PCR samples underwent purification using an 8% polyacrylamide gel. The DNA segments within the range of 140–160 base pairs (including the size of short noncoding RNA and the 3′ and 5′ adaptors) were retrieved and dissolved in 8 μL of elution solution. The assessment of library quality was conducted by utilizing the Agilent Bioanalyzer 2100 system and employing DNA High Sensitivity Chips.

### 2.4 Clustering and sequencing (Novogene Co., Ltd.)

According to the instructions provided by the manufacturer, the index-coded samples were clustered using the TruSeq SR Cluster Kit v3-cBot-HS (Illumina, Inc., San Diego, CA, United States) on a cBot Cluster Generation System. Following the creation of clusters, the libraries underwent sequencing on an Illumina Hiseq 2500/2000 platform, resulting in the generation of 50-bp single-end reads.

### 2.5 Sequence analysis

The DESeq R package (1.8.3) was used to analyze differential expression between two conditions/groups for samples that had biological replicates. The adjusted *P*-values were calculated using the Benjamini & Hochberg approach. By default, a threshold of 0.05 was established as the corrected *p*-value for determining significant differential expression. The DEGseq (2010) R package was used to conduct the differential expression analysis for the samples that did not have biological replicates. The q-value was utilized to adjust the *P*-value. The default threshold for significantly differential expression was set as q-value <0.01 and |log2(foldchange)|>1. The target gene of miRNA was predicted using psRobot tar in miRanda (Storey, 2003).

### 2.6 Quantitative RT-PCR

The Gene Amp PCR System 9700 (Applied Biosystems) was utilized to perform quantitative RT-PCR (qRT-PCR). Table 2 contains the PCR primers.

**TABLE 2:** Primers for the miRNAs.

Gene	RT primer
U6	5′CGC​TTC​ACG​AAT​TTG​CGT​GTC​AT3′
hsa-miR-99a-5p	5′GTC​GTA​TCC​AGT​GCG​TGT​CGT​GGA​GTC​GGC​AAT​TGC​ACT​GGA​TAC​GAC​CAC​AAG3′
hsa-miR-127-3p	5′GTC​GTA​TCC​AGT​GCG​TGT​CGT​GGA​GTC​GGC​AAT​TGC​ACT​GGA​TAC​GAC​AGC​CAA3′
hsa-miR-136-3p	5′GTC​GTA​TCC​AGT​GCG​TGT​CGT​GGA​GTC​GGC​AAT​TGC​ACT​GGA​TAC​GAC​AGA​CTC3′
hsa-miR-140-5p	5′GTC​GTA​TCC​AGT​GCG​TGT​CGT​GGA​GTC​GGC​AAT​TGC​ACT​GGA​TAC​GAC​CTA​CCA3′
hsa-miR-199b-5p	5′GTC​GTA​TCC​AGT​GCG​TGT​CGT​GGA​GTC​GGC​AAT​TGC​ACT​GGA​TAC​GAC​GAA​CAG​A3′
hsa-miR-202-5p	5′GTC​GTA​TCC​AGT​GCG​TGT​CGT​GGA​GTC​GGC​AAT​TGC​ACT​GGA​TAC​GAC​CAA​AGA3′
hsa-miR-181a-5p	5′GTC​GTA​TCC​AGT​GCG​TGT​CGT​GGA​GTC​GGC​AAT​TGC​ACT​GGA​TAC​GAC​ACT​CAC3′
hsa-miR-542-3p	5′GTC​GTA​TCC​AGT​GCG​TGT​CGT​GGA​GTC​GGC​AAT​TGC​ACT​GGA​TAC​GAC​TTT​CAG3′
hsa-miR-411-5p	5′GTC​GTA​TCC​AGT​GCG​TGT​CGT​GGA​GTC​GGC​AAT​TGC​ACT​GGA​TAC​GAC​CGT​GCG3′
hsa-miR-181d-5p	5′ GTC​GTA​TCC​AGT​GCG​TGT​CGT​GGA​GTC​GGC​AAT​TGC​ACT​GGA​TAC​GAC​ACC​CAC3′
hsa-miR-3074-3p	5′GTC​GTA​TCC​AGT​GCG​TGT​CGT​GGA​GTC​GGC​AAT​TGC​ACT​GGA​TAC​GAC​CGG​TGC3′
hsa-miR-1285-3p	5′GTC​GTA​TCC​AGT​GCG​TGT​CGT​GGA​GTC​GGC​AAT​TGC​ACT​GGA​TAC​GAC​AGG​TCT3′
hsa-miR-146b-3p	5′GTC​GTA​TCC​AGT​GCG​TGT​CGT​GGA​GTC​GGC​AAT​TGC​ACT​GGA​TAC​GAC​CCA​GAA​C3′
hsa-miR-486-3p	5′GTC​GTA​TCC​AGT​GCG​TGT​CGT​GGA​GTC​GGC​AAT​TGC​ACT​GGA​TAC​GAC​ATC​CTG3′
hsa-miR-486-5p	5′GTC​GTA​TCC​AGT​GCG​TGT​CGT​GGA​GTC​GGC​AAT​TGC​ACT​GGA​TAC​GAC​CTC​GGG3′
hsa-miR-1246	5′GTC​GTA​TCC​AGT​GCG​TGT​CGT​GGA​GTC​GGC​AAT​TGC​ACT​GGA​TAC​GAC​CCT​GCT3′
hsa-miR-504-5p	5′GTC​GTA​TCC​AGT​GCG​TGT​CGT​GGA​GTC​GGC​AAT​TGC​ACT​GGA​TAC​GAC​GAT​AGA3′
hsa-miR-326	5′GTC​GTA​TCC​AGT​GCG​TGT​CGT​GGA​GTC​GGC​AAT​TGC​ACT​GGA​TAC​GAC​CTG​GAG3′

### 2.7 Gene ontology enrichment analysis

The differentially expressed miRNAs were subjected to gene ontology (GO) enrichment analysis, specifically focusing on their target gene candidates. The GO enrichment analysis was conducted using the Wallenius non-central hypergeometric distribution (Young et al., 2017) based on GO seq, which has the ability to account for bias in gene length.

### 2.8 KEGG pathway analysis

The KEGG, also known as Kyoto Encyclopedia of Genes and Genomes, serves as a valuable database for comprehending the overarching operations of biological systems using molecular-level data, particularly vast molecular datasets produced by genome sequencing and other advanced experimental technologies (Kanehisa et al., 2023). We utilized the KOBAS program to evaluate the statistical enrichment of the potential gene candidates in KEGG pathways (Mao et al., 2005).

### 2.9 qRT-PCR for predicted target genes

The relative variations in miRNA expression were standardized using endogenous miRNAs as a control. A comparison was made between data obtained from samples of patients with osteoporosis and those without osteoporosis using Student’s t-test.

### 2.10 Statistical analysis

The findings were displayed as averages plus standard deviations (SD). For statistical analysis, the Mann-Whitney U-test or Wilcoxon signed-rank test was used. Analysis was conducted using SPSS 21.0 software (IBM, Armonk, NY, United States). Statistically significant results were those with two-sided *P*-values less than 0.05.

## 3 Results

### 3.1 Differentially expressed miRNAs in patients with osteoporosis and non-osteoporosis

**FIGURE 1:**
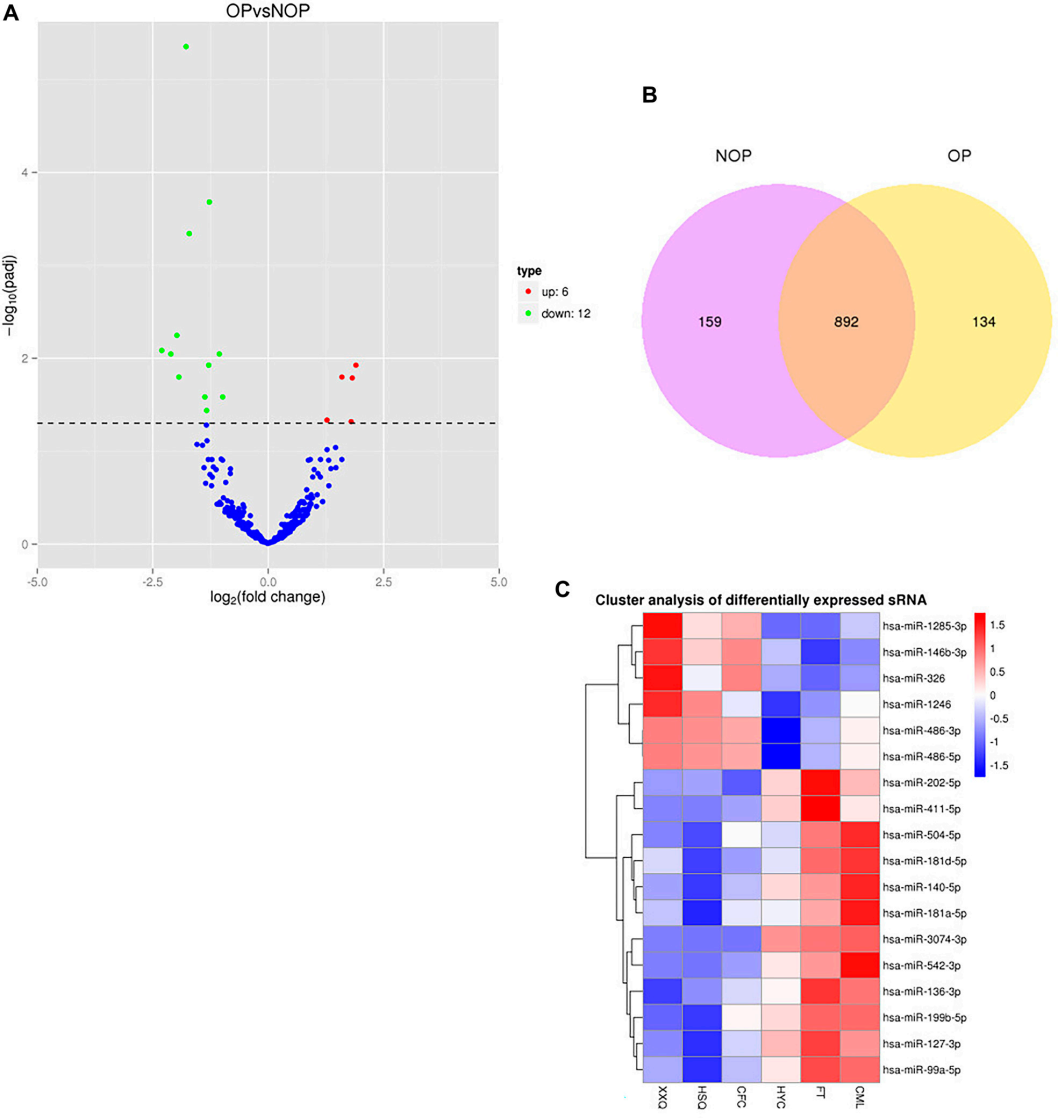
Differentially expressed miRNAs in patients with osteoporosis and non-osteoporosis. **(A)** The volcano plot was constructed using the *P*-values and fold change values. The abscissa represents the fold change in miRNA expression in the different experimental groups/different samples; the ordinate represents a statistically significant degree of changes in miRNA expression levels; the smaller the corrected *p*-value, the larger the -log10(corrected *p*-value), i.e., the more significant the difference. The scatter in the figure represents individual miRNAs, the blue circles represent miRNAs with no significant differences, and the red circles represent miRNAs with significant differences. **(B)** Venn diagram of putative targets from the TargetScan, Ingenuity, and miRNAs microarray analysis. **(C)** Clustered heat map showing the differentially expressed miRNAs between the two groups. Red indicates upregulated expression, and blue indicates down-regulated expression.

The overall distribution of the differential miRNAs can be inferred using volcano plots, which were obtained by screening the fold-change and adjusted significance levels. When the samples have biological repeats, the screening condition for differential miRNAs was Padj < 0.05. If the sample was not replicated biologically, there would be an excessive amount of distinct miRNAs. To manage the rate of false positives, the *p*-values were merged with the fold-changes. The criteria for screening the differentially expressed miRNAs were a q-value less than 0.01 and an absolute value of log2 (fold change) greater than 1. After screening the genes, they were statistically summarized, and a Venn diagram was created to show the counts of genes that were expressed and the counts of genes expressed in common (Figure 1A). Figure 1B and Supplementary Figure S1 showed a total of 12 miRNAs that were downregulated and five miRNAs that were upregulated. Clustering analysis of differentially expressed miRNAs was employed to identify the clustering patterns of the various miRNAs across distinct experimental conditions. Hierarchical cluster analysis, k-means cluster analysis, and SOM cluster analysis were performed using the TPM values of the union of all comparison combinations of distinct miRNA sets in each experimental group/sample, resulting in different miRNA sets for each comparison combination (Figure 1C).

### 3.2 Functional annotation of osteoporosis-associated miRNAs

**FIGURE 2:**
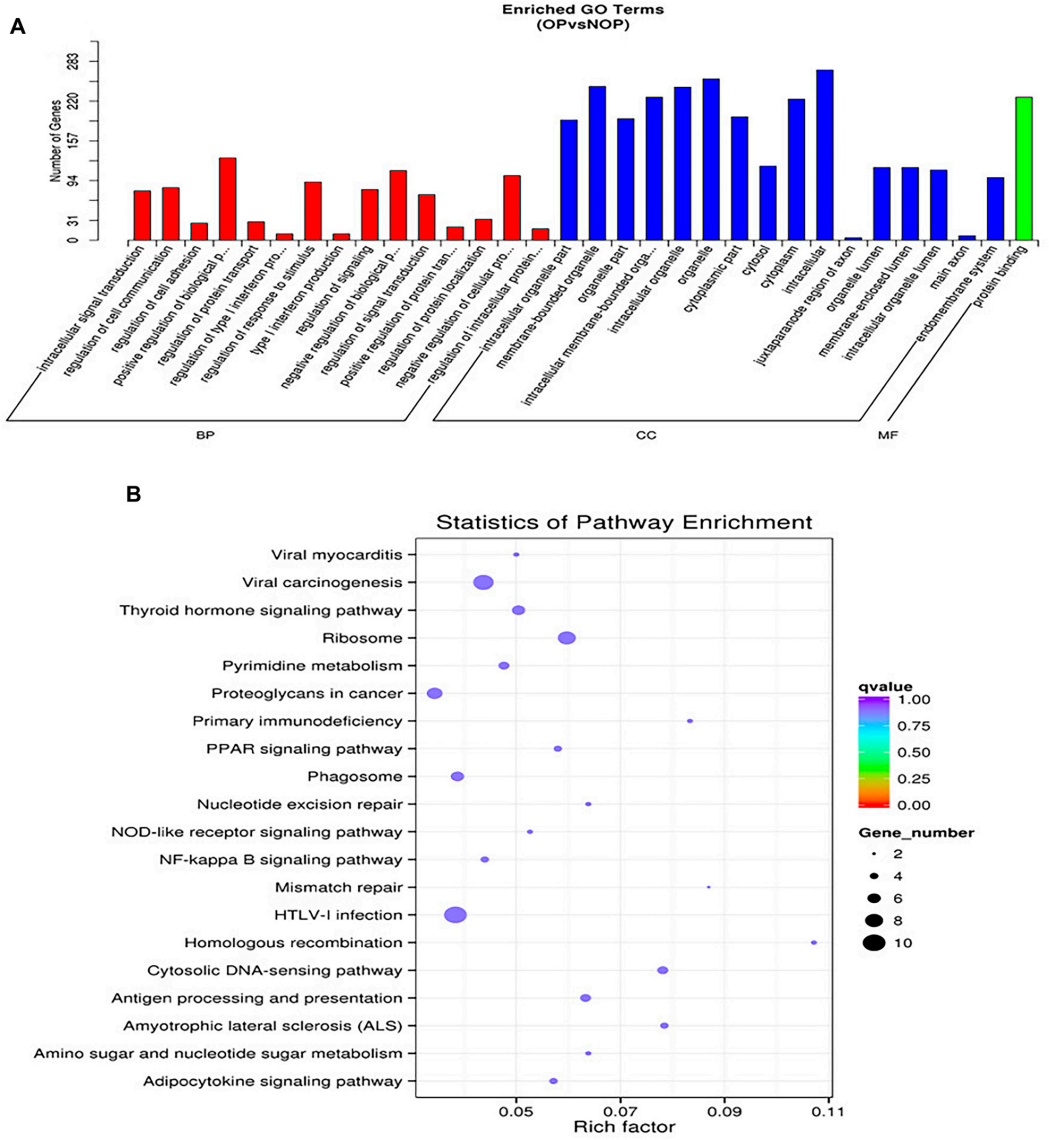
Functional annotation of osteoporosis associated miRNAs. **(A)** Representative gene ontology (GO) enrichment analysis. BP: top 10 enriched GO terms, describing the biological processes. CC: top 10 enriched GO terms describing cellular components. MF: top 10 enriched GO terms describing molecular functions. **(B)** Representative KEGG pathway enrichment analysis for osteoporosis and non-osteoporosis.

Following the comparison of differentially-regulated miRNAs among groups, we conducted GO and KEGG analyses on the target gene sets for each group of miRNAs, considering their corresponding relationship. To facilitate explanation, the collection of genes targeted by miRNAs with differential expression is denoted as “potential target genes” (Figure 2A). Based on their relationship, the three categories of GOs have the ability to create directed acyclic graphs. This structure shows the links between the GO nodes (a, part of, regulate, etc.).Figure 2B illustrates the diverse colors used to indicate various enrichment levels of GO in the KEGG pathway enrichment analysis, showcasing the biological importance of the research subject.

### 3.3 Validation of osteoporosis-associated miRNAs

**FIGURE 3:**
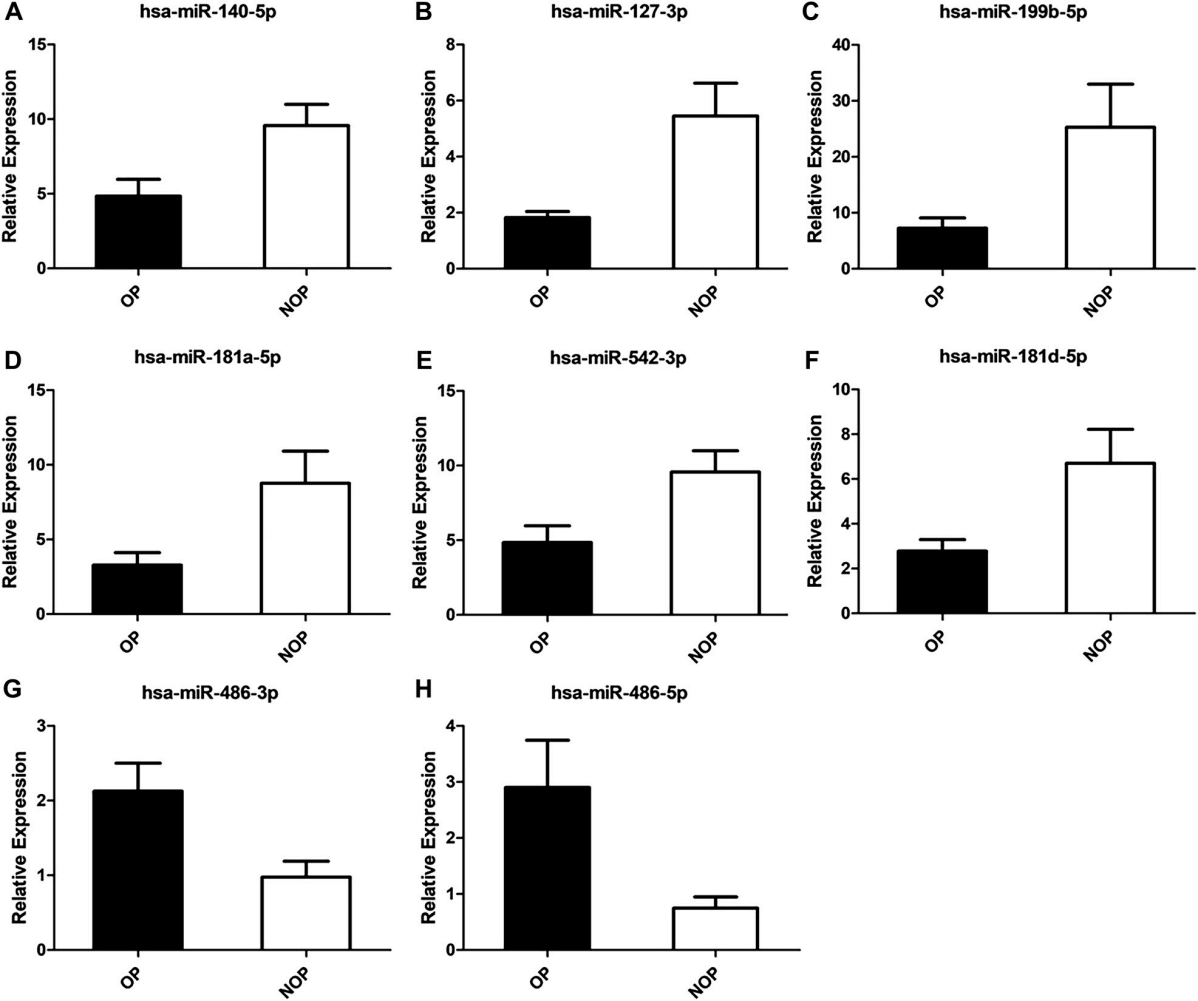
Validation of osteoporosis associated miRNAs. **(A)** has-miR-140-5p. **(B)** has-miR-127-3p. **(C)** has-miR-199b-5p. **(D)** has-miR-181a-5p. **(E)** has-miR-542-3p. **(F)** has-miR-181d-5p. **(G)** has-miR-486-3p. **(H)** has-miR-486-5p.

The validation of differentially-expressed miRNAs was performed using qRT-PCR, including miR-99a-5p, miR-127-3p, miR-136-3p, miR-140-5p, miR-199b-5p, miR-202-5p, miR-181a-5p, miR-542-3p, miR-411-5p, miR-181d-5p, miR-3074-3p, miR-1285-3p, miR-146b-3p, miR-486-3p, miR-486-5p, miR-1246, miR-504-5p, and miR-326. The selection of these miRNAs was based on their ranking in the list of miRNAs with differential expression and their predicted importance in bone growth and development. The qRT-PCR data confirmed that the expression of miR-140-5p, miR-127-3p, miR-199b-5p, miR-181a-5p, miR-181d-5p, and miR-542-3p was differentially regulated in osteoporosis compared to control specimens. The expression of these miRNAs was decreased by 2.27-, 3.00-, 3.48-, 2.67-, 2.41-, and 1.98-fold, respectively (all *P* < 0.05). On the other hand, miR-486-3p and miR-486-5p were upregulated by 2.17- and 3.89-fold (*P* < 0.05) (Figure 3). According to qRT-PCR, the outcomes of the remaining 10 miRNAs that exhibited no significant disparities between the two groups.

## 4 Discussion

Osteoporosis is a major health issue. miRNAs have various functions in controlling cell proliferation and the process of growth and maturation. The screening and validation of miRNAs related to osteoporosis were conducted using high-throughput sequencing. In osteoporosis, the findings indicated that six miRNAs (miR-140-5p, miR-127-3p, miR-199b-5p, miR-181a-5p, miR-181d-5p, and miR-542) exhibited decreased expression, whereas two miRNAs (miR-486-3p and miR-486-5p) displayed increased expression. The initial manifestation of various miRNAs might serve as predictive indicators and potentially anticipate the progression of osteoporosis.

In recent times, there has been a concentrated endeavor to thoroughly examine the alterations observed in the genetic material, epigenetic markers, RNA molecules, and protein profiles in osteoporosis. The aim is to identify novel genes responsible for driving osteoporosis, which could potentially have clinical implications. Exploring novel treatments for osteoporosis can be achieved by identifying previously undiscovered genes associated with the condition. During both normal development and diseases (Diener et al., 2022), miRNAs play a crucial role in controlling various processes such as cell-fate determination, proliferation, differentiation, and apoptosis. The high-throughput sequencing results of patients with osteoporosis and patients without osteoporosis were analyzed using hierarchical clustering, GO analysis, KEGG analysis, and network regulation analysis. Significant variations in the expression of multiple miRNAs were observed when comparing the groups.

Osteoarthritis has been linked to MicroRNA-140. Miyaki et al. conducted a study in the year 2009. A gene expression profiling experiment was conducted by (Miyaki et al., 2009; Miyaki et al., 2010) using miRNA microarrays and qRT-PCR to compare human articular chondrocytes with human mesenchymal stem cells (MSCs). According to the microarray analysis, chondrocytes and MSCs exhibited the most significant variation in miR-140 expression. MiR-140 was expressed in healthy human articular cartilage, but its expression was notably diminished in tissue affected by osteoarthritis. Subsequently, a research article was released to investigate the functions of microRNA-140 in both cartilage development and homeostasis, as stated in reference (Zhang et al., 2012). The study demonstrated that miR-140 was present in every sample of knee synovial fluid, with its expression significantly reduced in the osteoarthritis group compared to the control group. During endochondral bone development, miR-140 exhibited significant involvement (Papaioannou et al., 2013; Papaioannou et al., 2015). The expression of MEF2C is reduced by the inhibition of p38 MAPK signaling, which is negatively controlled by miR-140 (Khiem et al., 2008). Mice lacking miR-140 exhibited two distinct characteristics: firstly, the absence of miR-140 during the initial phases of bone formation, specifically when the hyaline cartilage framework is established; and secondly, a temporary localization of miR-140 in the core of the bone, coinciding with the primary ossification center (Nakamura et al., 2011). The findings suggest a novel function of miR-140 during the initial phases of endochondral ossification. Evidence is still lacking about whether miR-140 is related to osteogenesis. miR-140-5p exhibited decreased expression in individuals with osteoporosis, as observed in this research.

Bakhshandeh et al. (2012a); Bakhshandeh et al. (2012b), the modulation of various osteo-miRNAs (e.g., miR-199b, miR-1274a, and miR-30b) with shared targets (e.g., BMPR, TCFs, and SMADs) was demonstrated, indicating their role as mediators of osteogenic pathways involving cell-cell interactions, Wnt, and TGF-β signaling. This suggests a potential mechanism for the quick initiation of osteogenesis through anti-miRNA therapy. The expression of miR-199b varied among the exosomal samples obtained from cultured human bone marrow stem cells (hBMSCs) at different time intervals. DIANA-mirPath’s bioinformatic analysis revealed that quantiles exhibiting differential exosomal miRNA patterns associated with osteogenic differentiation were enriched in prominent pathways such as RNA degradation, mRNA surveillance pathway, Wnt signaling pathway, and RNA transport. miR-199b is an impact factor of osteoblast differentiation (Xu et al., 2014). The initial finding revealed that miR-199b-5p has a positive effect on the differentiation of human osteoblasts (Zhao et al., 2016). The study revealed that miR-199b-5p has an indirect effect on the regulation of cellular senescence in MSCs. Furthermore, differences specific to senescence were observed in the miRNA composition of small extracellular vesicles (sEVs), including a decrease in miR-199b-5p levels (Yoo et al., 2014; Terlecki-Zaniewicz et al., 2018). The study found that the level of miR-199b-5p expression was reduced in individuals with osteoporosis, suggesting that miR-199b could potentially have a significant impact on the regulation of bone metabolism through its involvement in cellular senescence.

Next-generation sequencing and quantitative reverse-transcription PCR (qRT-PCR) were employed to analyze the miRNA expression profiles in human serum samples collected from individuals of different age groups, with a mean age of 30 years for the young group and 64 years for the older group (Noren et al., 2013). Among the miRNAs identified in the serum, miR-181a-5p exhibited a notable decline in 20 elderly participants in comparison to 20 younger individuals. The implication is that miR-181a-5p could potentially serve as a biological indicator of the aging process. B-cell lymphoma-2 (Bcl-2) is targeted by miR-181a-5p and miR-181d-5p (Ouyang et al., 2012; Wang et al., 2012). Furthermore, the examination of microarray and qRT-PCR indicated that miR-181a-5p and miR-181d-5p exhibited significant upregulation in normally healing fractures in contrast to non-healing fractures (Waki et al., 2016). Regarding the relationship between BCL2 and miR-181a-5p, it has been shown that both exhibit similar trends of change under the same intervention conditions. However, depending on the change in the level of miR-181d-5p in the exosomes, the effect caused by its targeted binding to BCL2 may show different trends (Yadav et al., 2022; Chai et al., 2024). Interestingly, the current investigation revealed that miR-181a-5p and miR-181d-5p exhibited decreased expression levels in individuals with osteoporosis, indicating potential significance of these two miRNAs in the process of bone remodeling. Apoptosis is induced in both the mitochondrial pathway and the death receptor pathway when the p53 protein becomes activated and reaches a specific threshold. This activation leads to the transcriptional activation of pro-apoptotic genes like Bax, p53 upregulated modulator of apoptosis (PUMA), and Fas receptor. Additionally, it transcriptionally inhibits anti-apoptotic genes such as Bcl-2. According to Moriishi and colleagues, the study (Moriishi et al., 2011) demonstrated that the excessive expression of Bcl2 in osteoblasts hinders the differentiation of osteoblasts, diminishes osteocyte processes, and leads to osteocyte apoptosis.

The study demonstrated that miR-542-3p plays a pivotal role as a novel enhancer of p53 (Wang et al., 2018). According to (Kureel et al., 2014), it was proposed that the excessive presence of miR-542-3p hindered the differentiation of osteoblasts. Conversely, the suppression of miR-542-3p’s role through anti-miR-542-3p encouraged the manifestation of genes specific to osteoblasts, activity of alkaline phosphatase, and mineralization of the matrix. Moreover, their findings strongly indicated that miR-542-3p inhibits the process of bone formation and enhances the programmed cell death of bone-forming cells by suppressing BMP-7 and its subsequent signaling pathway. Furthermore, a notable inverse association was observed between the expression of miR-542-3p messenger RNA in rats that underwent ovariectomy (Zhang et al., 2018). The analysis of microarray data also indicated that miR-542-3p exhibited differential expression in the bone marrow cells of rats that underwent ovariectomy (Semeghini et al., 2018). The findings of our study indicated a decrease in the expression of miR-542-3p in individuals with osteoporosis. Considering that miR-542-5p is regulated through p53 signaling and the p53 gene influences the activity of Bcl2 family genes, it is likely that bone metabolism will be impacted.

Skeletal muscle is where MiR-127 is commonly expressed. The expression of miR-127 increases during the differentiation of C2C12 and satellite cells (SC). Enhancing myogenic cell differentiation in C2C12 cells is achieved through the overexpression of miR-127 (Li et al., 2016). It was proposed that miR-127-3p likely plays a role in the growth and specialization of myoblasts, which further contributes to the regulation of muscle characteristics in goats.

Despite not having a muscle-specific expression, miR-486, the most recent addition to the myomiR group, plays a crucial role in skeletal muscle development processes. According to the report, miR-486 exhibited significant upregulation throughout the process of muscle differentiation, specifically targeting PAX7 and consequently expediting the differentiation of myoblasts (Dey et al., 2011). Furthermore, in dystrophic muscle, the decreased level of miR-486 leads to significant improvements in the physiological function and strength of the muscle when miR-486 is overexpressed (Alexander et al., 2014). Result: Conversely, the study demonstrated that sarcopenia is associated with reduced bone mineral density (BMD) and osteoporosis in middle-aged and older men. In osteoporosis, the study revealed an increase in miR-486-3p and miR-486-5p expression, whereas miR-127-3p expression was decreased. Exploring the connection between muscle and bone, specifically sarcopenia and osteoporosis, can offer a fresh perspective. It is an interesting subject of research.

There are constraints to this study. The study was conducted on a limited number of participants, who all shared the same ethnic and genetic heritage. Solely bone tissues were analyzed, and forthcoming research should encompass a wider range of tissue types. Confirmation of these findings will be necessary in subsequent research.

## 5 Conclusion

To sum up, osteoporosis resulted in the downregulation of miR-140-5p, miR-127-3p, miR-199b-5p, miR-181a-5p, miR-181d-5p, and miR-542-3p, whereas miR-486-3p and miR-486-5p were upregulated. This study identified osteoporosis-specific miRNAs and suggested some evidence of relationships between muscles and bones. The findings of this research can offer fresh information to support the advancement of innovative treatments and/or preventive strategies for osteoporosis.

## Data Availability

The data presented in the study are deposited in the GEO repository, accession number GSE273345.

## Supplementary material

SUPPLEMENTARY FIGURE S1 Differentially expressed miRNAs in patients with osteoporosis and non-osteoporosis.

## Abbreviations

CI, Confidence interval; GO, Gene ontology; KEGG, Encyclopedia of Genes and Genomes; miRNAs, MicroRNAs; qRT-PCR, Quantitative RT-PCR; RISC, RNA-induced silencing complex.
