# Supplementary figures and images for: Identification of miRNAs related to osteoporosis by high-throughput sequencing
Source: Front Pharmacol. 2024 Aug 8;15:1451695. doi: 10.3389/fphar.2024.1451695 (PMC11338934; doi:10.3389/fphar.2024.1451695)

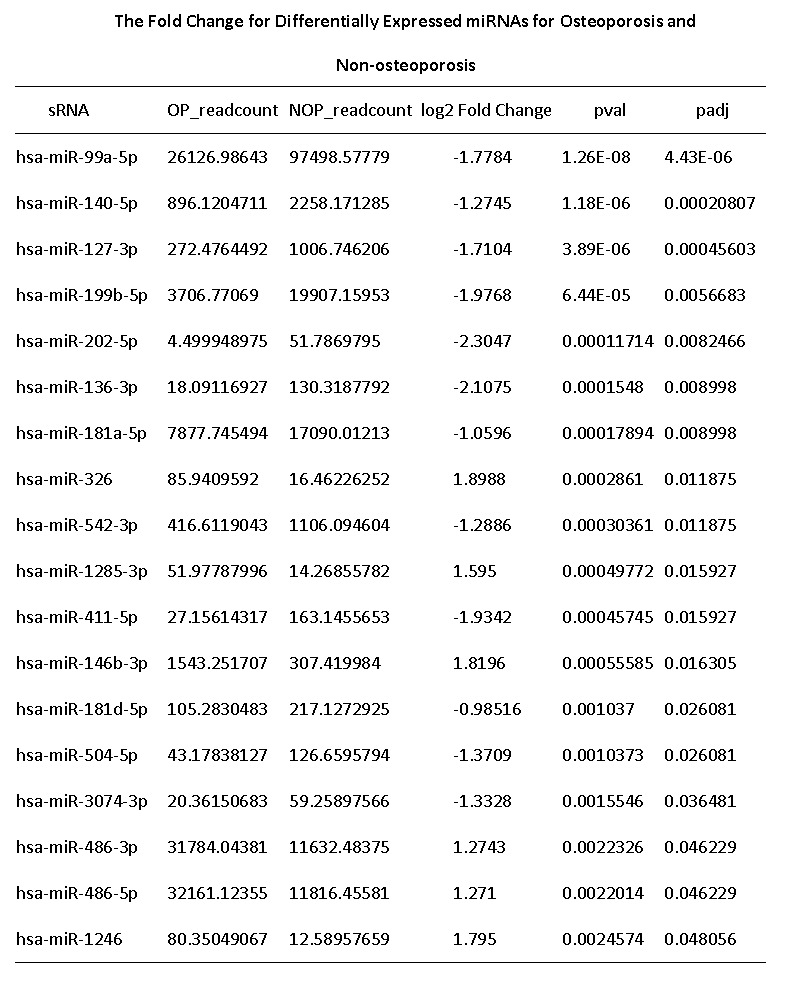

Supplement: Supplementary file 1 [file Image1.jpeg]
